# Supplementary material for: Transcriptional and Chromatin Accessibility Profiling of Neural Stem Cells Differentiating into Astrocytes Reveal Dynamic Signatures Affected under Inflammatory Conditions
Source: Cells. 2023 Mar 21;12(6):948. doi: 10.3390/cells12060948 (PMC10047363; doi:10.3390/cells12060948)

Supplementary Figure S1

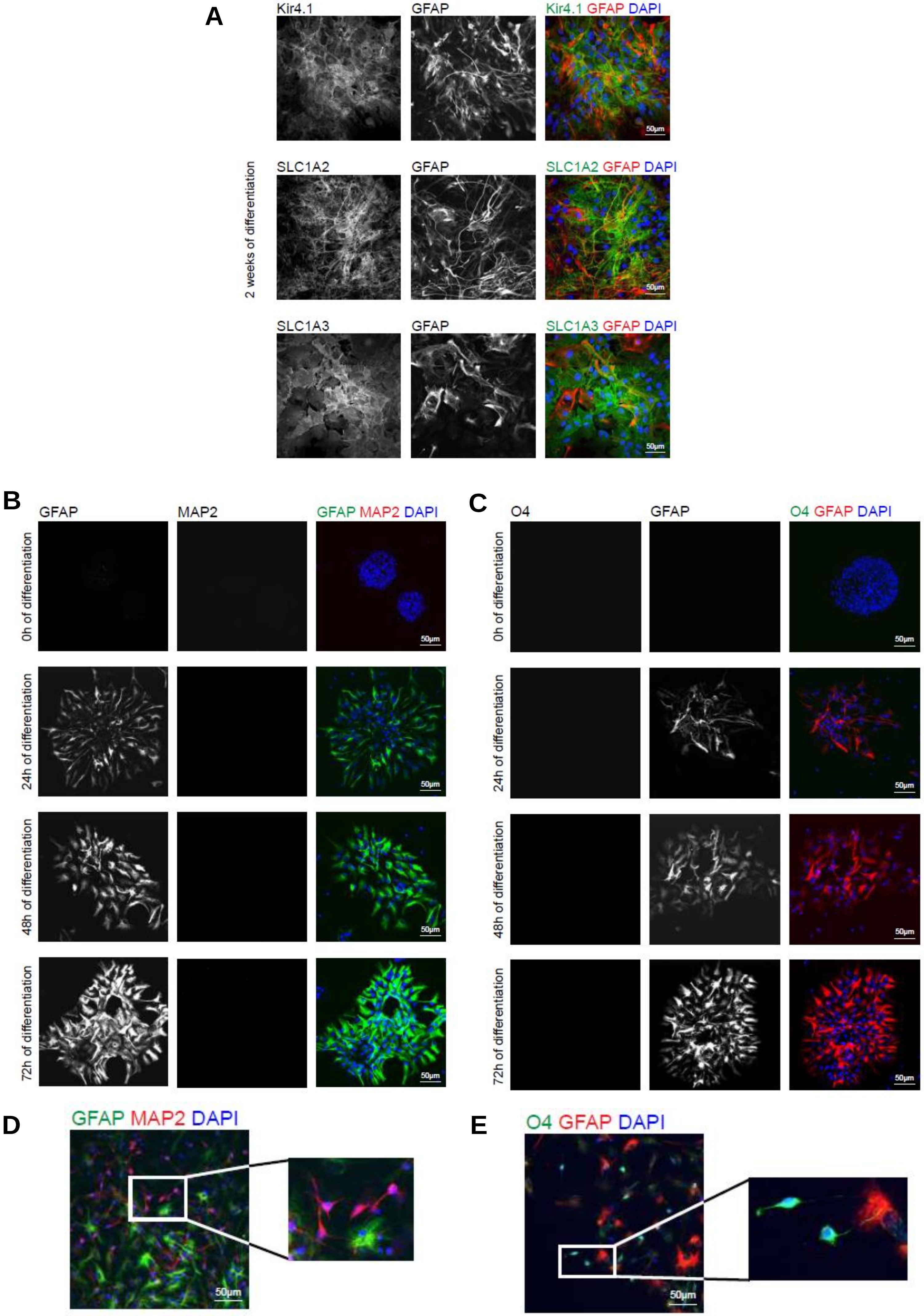

Supplementary Figure S2

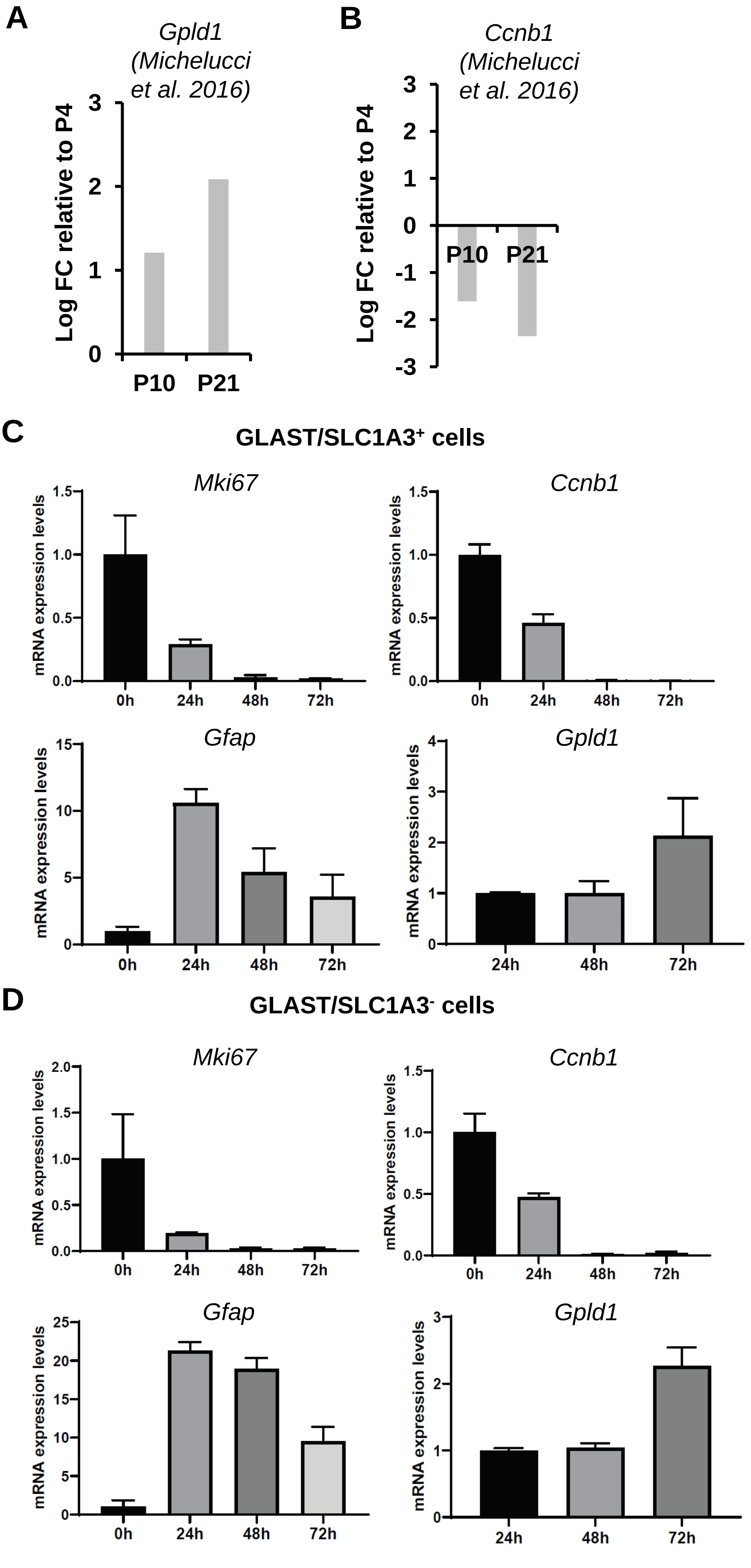

Supplementary Figure S3

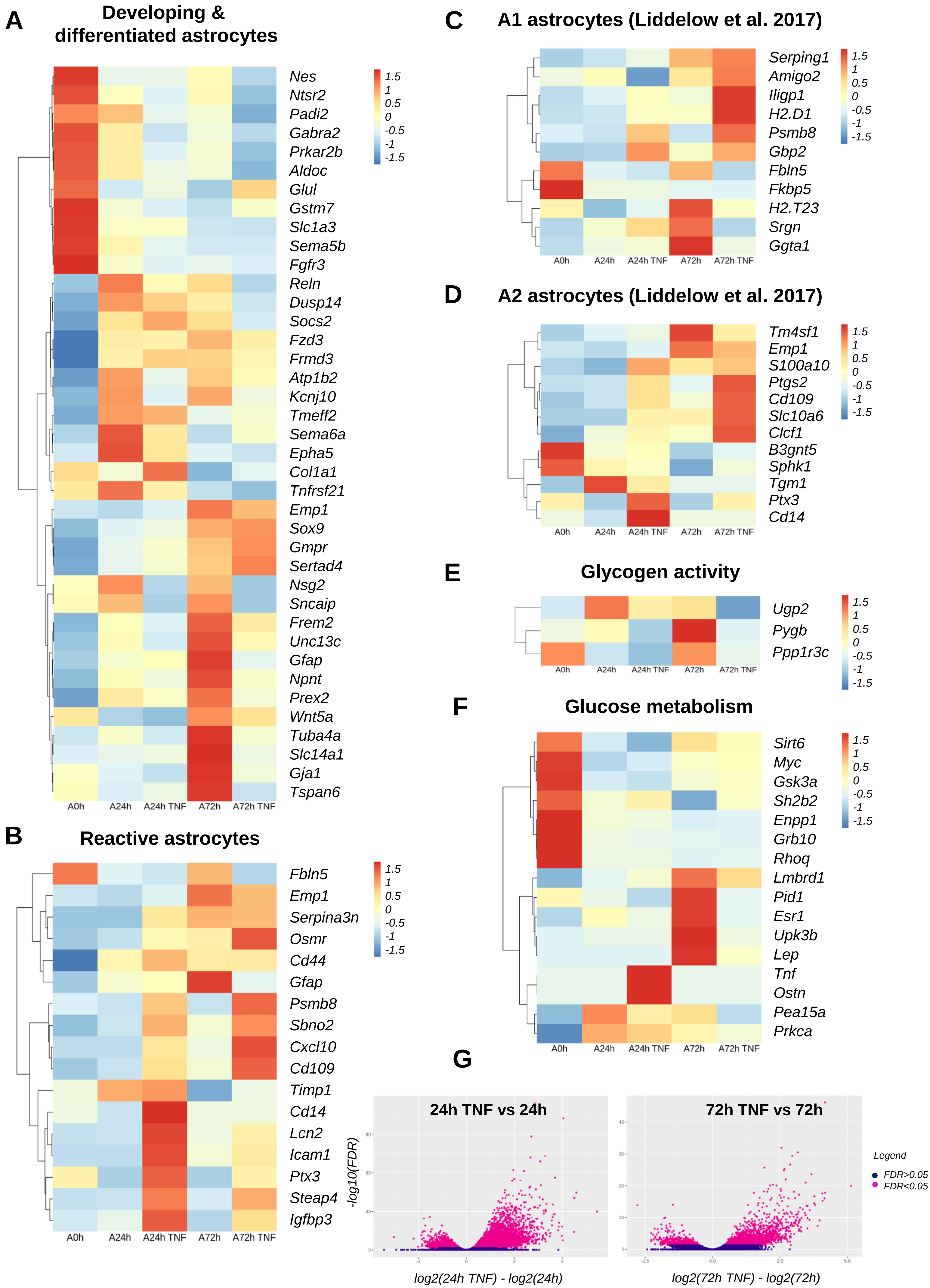

Supplementary Figure S4

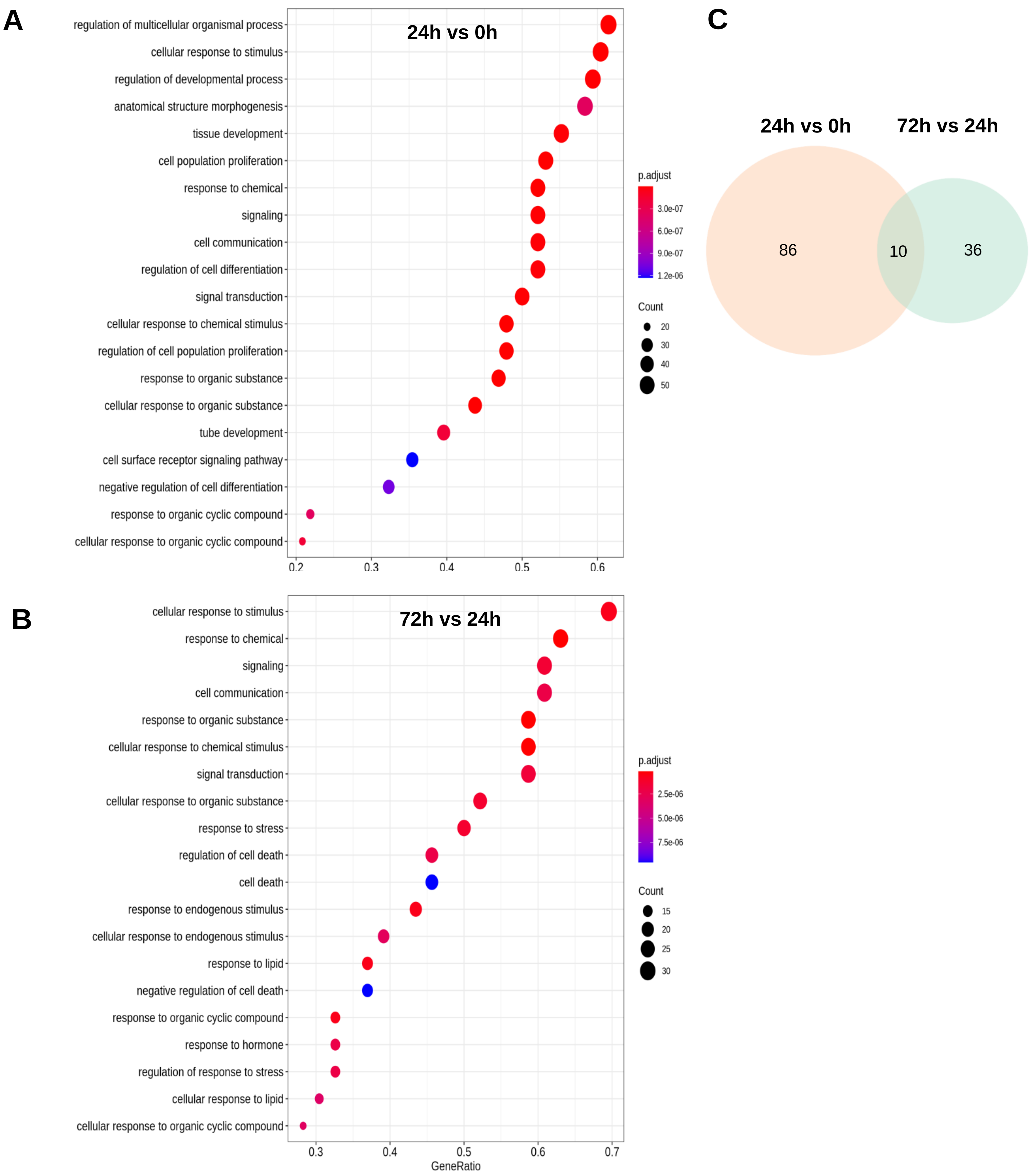

Supplementary Figure S5

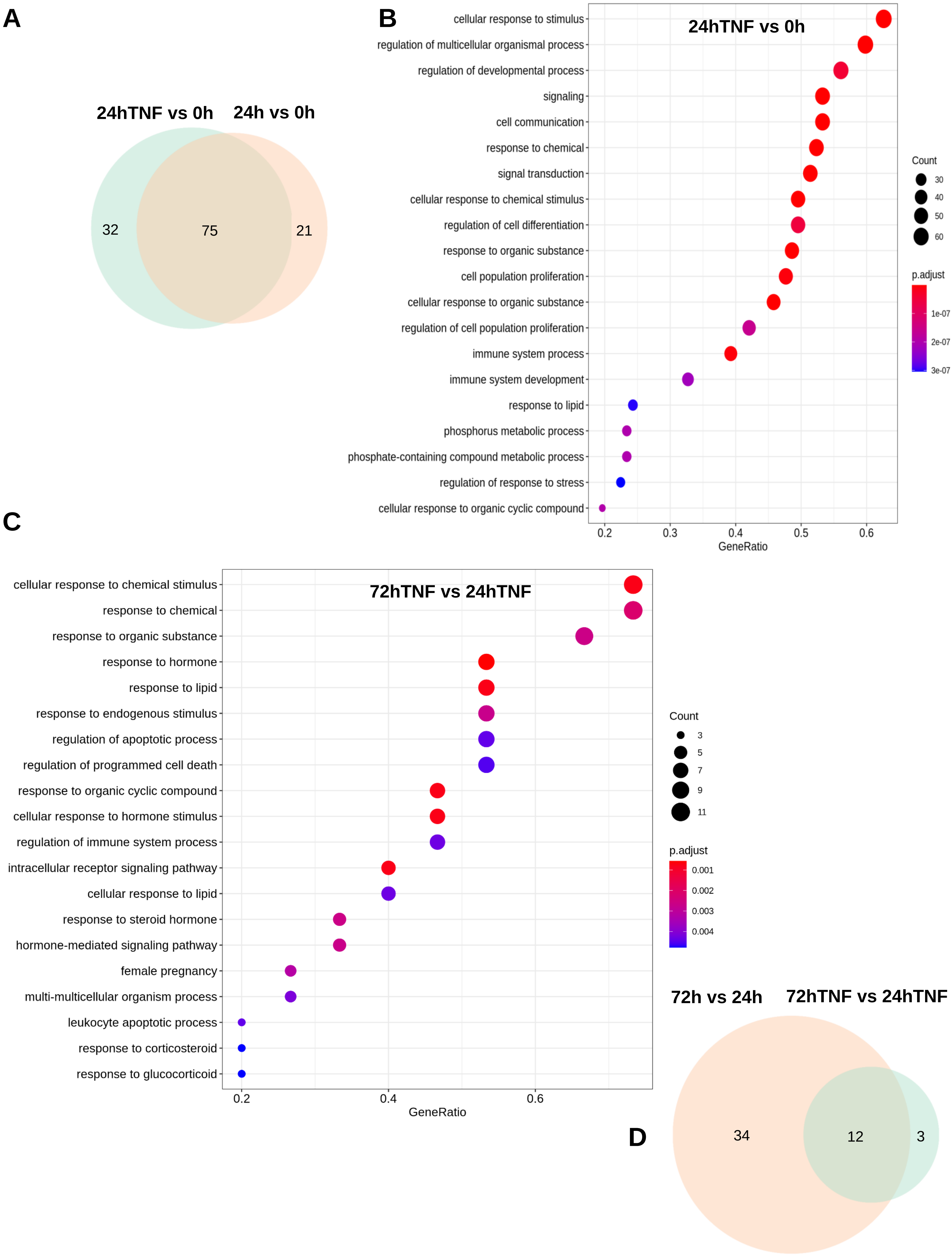

Supplement: Supplementary file 1 [file cells-12-00948-s001.zip › cells-2199691-supplementary.pdf]
